# Vintage Victory: Warfarin Versus Apixaban in the Antiphospholipid Arena

**DOI:** 10.7759/cureus.57040

**Published:** 2024-03-27

**Authors:** Michael Sabina, Zein Barakat, Bernardo Costa Guerra, Andrew Lurie, Zoya Khan

**Affiliations:** 1 Internal Medicine, Lakeland Regional Health Medical Center, Lakeland, USA; 2 Research, Nova Southeastern University Dr. Kiran C. Patel College of Osteopathic Medicine, Davie, USA; 3 Research, Lakeland Regional Health Medical Center, Lakeland, USA

**Keywords:** thrombosis management, direct oral anticoagulants (doacs), apixaban, warfarin, antiphospholipid syndrome (aps)

## Abstract

This case report explores the efficacy of warfarin compared to apixaban in managing antiphospholipid syndrome (APS), an autoimmune disorder characterized by recurrent thrombosis. We emphasize the constraints of direct oral anticoagulants (DOACs) such as apixaban in APS management. This case discusses a 41-year-old female patient with APS who did not respond to apixaban therapy. The report details her transition to warfarin, resulting in symptom resolution and no further complications, thus alluding to warfarin's effectiveness in APS management over apixaban. The case contributes to the ongoing debate on the suitability of modern DOACs in APS treatment.

## Introduction

Antiphospholipid syndrome (APS) is an autoimmune disorder characterized by recurrent thrombosis in the setting of elevated antiphospholipid antibodies [1,2]. Common clinical manifestations include recurrent venous or arterial thrombosis, thrombotic events in unusual locations, and obstetric complications such as recurrent early fetal loss and pre-eclampsia [1,2]. Management of APS requires long-term anticoagulation to mitigate thrombotic risks. Potential options include vitamin K antagonists (VKAs) and direct oral anticoagulants (DOACs), each targeting distinct aspects of the coagulation cascade. VKAs target vitamin K-dependent factors, II, VII, IX, and X, thereby affecting the intrinsic and extrinsic pathways directly [3]. Apixaban reversibly inhibits factor Xa, preventing the conversion of prothrombin to thrombin and thus impacting the common coagulation pathway [4]. DOACs offer advantages over VKAs, including fixed-dose prescribing, fewer dietary restrictions, reduced major intracranial bleeding, and significantly fewer drug-drug interactions [4,5]. Despite these advantages, conflicting recommendations exist regarding the use of DOACs in APS management. The American Society of Hematology guidelines recommend against the use of DOACs in all APS patients, while the International Society on Thrombosis and Haemostasis advises caution when considering DOACs for APS, especially in high-risk profiles, advocating VKAs as the first-line treatment with a targeted international normalized ratio (INR) of 2.0-3.0 [4,5]. We present a case of a patient with APS who failed anticoagulation treatment with apixaban, a direct factor Xa inhibitor, and discuss our decision for the alternative with a VKA, namely warfarin.

## Case presentation

**Figure 1:**
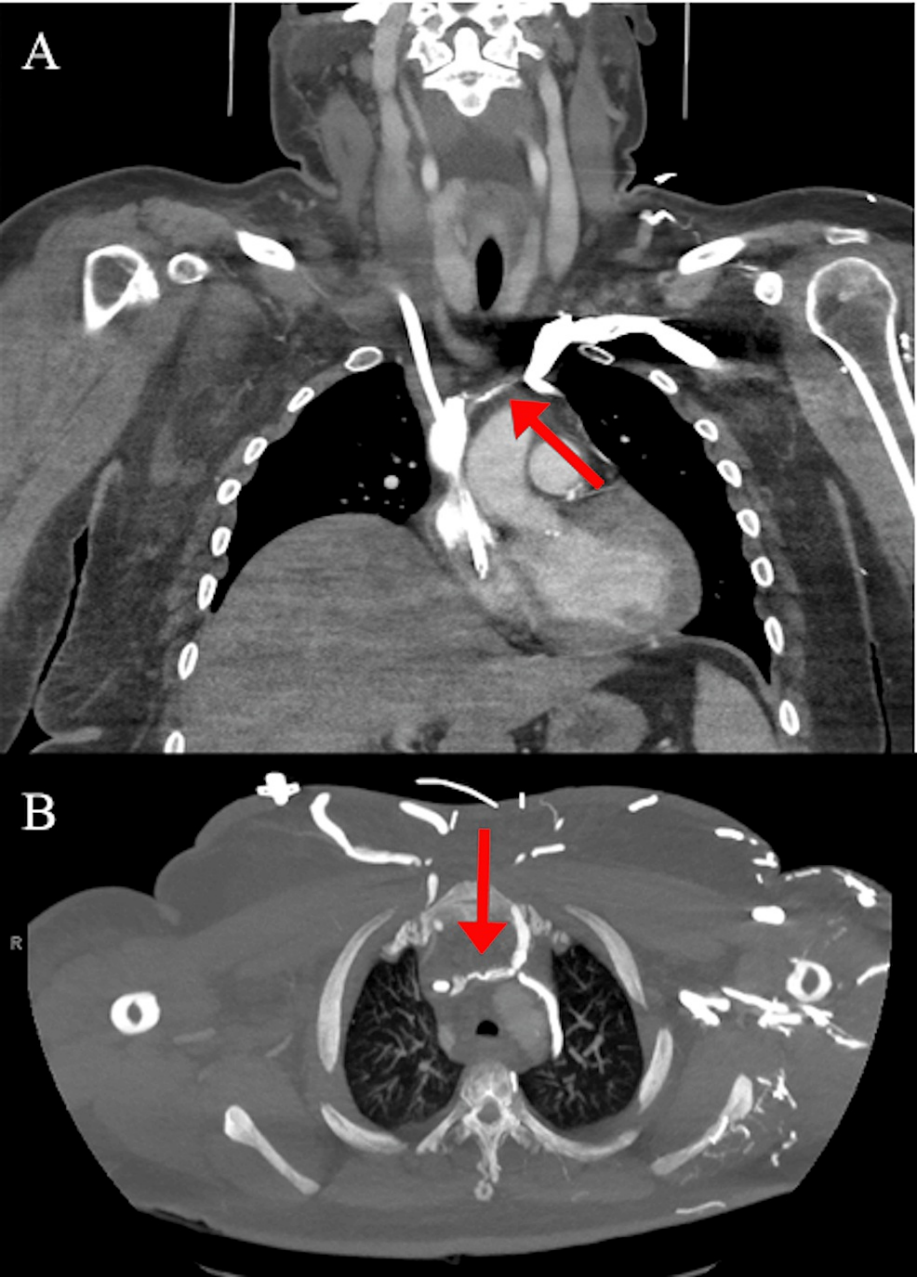
Computed tomography (CT) scan of chest and neck with brachiocephalic vein occlusion (red arrows)

This is a female in her 40s with a past medical history significant for pulmonary embolism and deep vein thrombosis (DVT) on apixaban, Sjögren's syndrome, hypertension, and pre-eclampsia with resultant postpartum end-stage renal disease on dialysis. She presented to the hospital for marked bilateral swelling and pain in her right arm, face, and neck that has been slowly progressing over the course of the past month. Vital signs were within normal limits and there were no other concerning symptoms such as angina, dyspnea, shortness of breath, or fever present. Laboratory work was significant only for creatine of 2.99 mg/dL and a glomerular filtration rate of 20 ml/min/1.73m^2 coinciding with her baseline renal function. Workup with a computed tomography scan revealed occlusions of the brachiocephalic veins near the site of her vas cath (Figure 1).

Initial management included discontinuation of apixaban and initiation of continuous heparin infusion. Upon further questioning, it was learned of a family history of systemic lupus nephritis in her mother. Given the peculiar presentation of clot formation with failure of outpatient anticoagulation therapy, this prompted a hypercoagulability workup, which revealed a positive test for antinuclear antibody and anti-phosphatidylserine/prothrombin complex IgM antibodies.

The patient was started on warfarin due to the failure of apixaban therapy and optimized with a target INR of 2.0-3.0 upon discharge. The patient routinely follows up with our outpatient clinic, which we have been following for six months after discharge. Her symptoms of upper extremity, face, and neck swelling have resolved. INR has been stable in the goal range for six months, with no further complications of DVT, PE, or signs of thrombosis.

## Discussion

We observed an instance where apixaban, a DOAC, failed to prevent thrombotic complications in a patient with APS. This case demonstrates the limitations of apixaban. Despite its known advantages, the patient developed occlusions in her brachiocephalic veins. After converting over to warfarin and following up with us for six months, the patient has not had any thrombotic events or complications. This case highlights the necessity of closely examining the efficacy of apixaban in APS management. Modern oral anticoagulants such as apixaban and rivaroxaban have revolutionized anticoagulation therapy. These DOACs provide a convenient alternative to warfarin by eliminating the need for dietary restrictions and routine monitoring. Their rapid onset of action, predictable pharmacokinetics with fixed-dose prescribing, and fewer drug interactions have made them a preferred choice for various indications [4]. Nevertheless, it is pivotal to compare the efficacy of DOACs to warfarin when considering a deviation from the established standard of care. The efficacy of rivaroxaban has been explored repeatedly with various levels of conflicting data. The RAPS trial found that APS patients treated with rivaroxaban had a significant increase in thrombin potential compared to warfarin users [6,7]. However, this trial primarily measured endogenous thrombin potential rather than clinical thrombosis frequency. Subsequent large, multicenter trials have failed to demonstrate rivaroxaban's noninferiority to warfarin in reducing thrombosis [3,8]. Apixaban, on the other hand, requires more research. A retrospective study comparing the incidence of thrombosis in APS patients using DOACs versus warfarin showed a threefold increase in thromboembolic events in the DOAC group, although not statistically significant [9]. Moreover, another retrospective study comparing DOACs to warfarin in the treatment of single antibody-positive APS exhibited similar incidence rates of thromboembolic events between the two groups, albeit not statistically significant [10]. Notably, apixaban constituted 40% of the DOAC group, while rivaroxaban accounted for 60%. The direct comparative efficacy of apixaban versus warfarin in APS patients remains relatively underexplored. A small randomized controlled trial comparing apixaban to warfarin in APS treatment yielded a notably lower incidence of thromboembolic events in the apixaban group, although the study was terminated early due to recruitment and funding challenges [11]. Thus, further exploration of this topic is essential, particularly through prospective randomized controlled trials covering a range of APS patient risk levels, which could provide valuable insights into the effectiveness and suitability of modern DOACs in APS management. This case aims to contribute to this ongoing discourse by offering valuable insights into the comparative efficacy of apixaban and warfarin in managing APS.

## Conclusions

In this case, warfarin versus apixaban in the management of APS was observed and presents compelling evidence for the effectiveness of warfarin in patients who do not adequately respond to apixaban. This case serves as a critical example and suggests the comparative effectiveness of traditional anticoagulants like warfarin against DOACs in APS; however, further research including randomized control trials is required to definitively make such a statement.
